# Efficacy and safety of D-penicillamine, trientine, and zinc in pediatric Wilson disease patients

**DOI:** 10.1186/s13023-024-03271-1

**Published:** 2024-07-09

**Authors:** Eun Joo Lee, Min Hyung Woo, Jin Soo Moon, Jae Sung Ko

**Affiliations:** 1 Department of Pediatrics, Severance Hospital, Yonsei University College of Medicine, Seoul, Korea; 2 Department of Pediatrics, Seoul National University Children’s Hospital, Seoul National University College of Medicine, Seoul, Korea

**Keywords:** Wilson disease, D-penicillamine, Trientine, Zinc, Pediatric

## Abstract

**Objectives:**

Wilson disease (WD) is a rare genetic disease affecting copper metabolism and the biliary tract’s copper excretion. Lifelong medication is necessary to prevent liver failure, neurological complications, and death. Although D-penicillamine (DPA), trientine, and zinc are used to treat WD, there is limited research on the long-term outcomes of these drugs, especially in children. This study aimed to evaluate the efficacy and safety of DPA, trientine, and zinc in patients diagnosed with WD during childhood.

**Methods:**

Ninety out of 92 patients were included in the analysis, excluding two patients who underwent liver transplantation without drug treatment due to an acute liver failure diagnosis. Treatment outcomes and reasons for discontinuation of therapy in 148 treatment blocks (37 DPA, 50 trientine, and 61 zinc) were analyzed using Kaplan–Meier analysis.

**Results:**

The median age at diagnosis was 8.3 years. There was a statistically significant difference in drug changes due to treatment ineffectiveness among the three drugs: trientine (22/50, 44%), zinc (15/61, 25%), and DPA (2/37, 5%) (all *p* < 0.05). Regarding drug changes due to adverse effects, the rate was the highest for DPA, followed by zinc and trientine. There were significant differences between DPA and zinc, zinc and trientine (all *p* < 0.05), but no significant difference was observed between DPA and zinc (*p* = 0.22).

**Conclusions:**

In pediatric WD, DPA, zinc, and trientine have therapeutic effects in that order. However, DPA and zinc are associated with more adverse effects compared to trientine.

## Introduction

Wilson disease (WD) is a genetic disease caused by mutations in ATP7B, an ATPase responsible for the excretion of copper from the liver into bile [1]. Initially, copper deposition occurs in the liver; however, as the disease progresses, copper accumulates in other organs, including the brain. The clinical symptoms of WD vary depending on the affected organ, leading to variations in the age at symptom onset. Hepatic symptoms typically manifest after 2 years of age, whereas neurological and psychiatric symptoms usually arise after the age of 10 years [2–6]. Recently, genetic testing has facilitated the diagnosis of WD, allowing the identification of asymptomatic individuals before 2 years of age [7]. Lifelong treatment is necessary to maintain a negative copper balance, as failure to do so can result in liver failure or neurological complications leading to death [8]. Treatment options consist of chelators, which enhance copper excretion in urine by eliminating copper deposits, and zinc, which inhibits copper absorption in the intestinal tract [9]. An adult study demonstrated that zinc monotherapy is less effective than chelator therapy in patients with declining liver function [10]; however, no relevant studies have been conducted in children. Moreover, there currently is no separate analysis of trientine in both pediatric and adult populations.

Although the therapeutic and adverse effects of WD drugs may vary among toddlers, adolescents, and adults, few studies have compared these drugs, particularly in children.

The present study aimed to compare the therapeutic and adverse effects of D-penicillamine (DPA), trientine, and zinc in children with WD.

## Methods

### Study population

We conducted a retrospective review of the charts of 92 patients diagnosed with WD at the Seoul National University Children’s Hospital from January 2005 to August 2021. The diagnosis of WD was based on clinical symptom, biochemical parameters and/or liver histological results and/or genetic analysis [11]. Baseline characteristics were collected at the time of diagnosis. The initial manifestations at the time of diagnosis were classified into three categories: hepatic, neurologic, and mixed presentation. Hepatic group included cases diagnosed through family screening or those with confirmed liver disease only, without neurological symptoms. Patients presenting with typical neurological and/or psychiatric symptoms without concurrent symptoms of chronic liver disease (jaundice, ascites, and edema) were classified as neurologic presentation; those patients who exhibit neurological and/or psychiatric symptoms along with symptoms of chronic liver disease were classified as mixed presentation.

Throughout the study period, two pediatric hepatologists treated patients with WD. The initial treatment doses were as follows: for DPA, the starting dose is 5 mg/kg/day or 150–300 mg/day, with an increase up to 20 mg/kg/day or 1000 mg (max 1500 mg); for trientine, 20 mg/kg/day or 1000 mg (max 1500 mg); and for zinc, the starting dose was 30 mg/kg for children under 6 years old, and 75 mg/day for those aged 6 or older. Urinary copper excretion of 200–500 µg/day and 30–75 µg/day were used as treatment parameters for DPA /trientine and zinc, respectively.

This study was approved by the Institutional Review Board (IRB) of the Seoul National University Children’s Hospital (IRB No. 1909–111-1066), Korea.

### Patient monitoring and treatment

Patients visited the outpatient clinic within 1 month of diagnosis, and the follow-up period was extended by 1–2 months thereafter. The follow-up period was set at 6 months when the patient’s LFT and symptoms remained stable. In cases where stability was not achieved, monitoring was performed at intervals of 2–4 months. If there was a change in treatment during the follow-up period, the reason for the change was investigated, which included factors such as treatment adverse effects, treatment ineffectiveness, drug unavailability, and patient’s request. Treatment ineffectiveness was considered when aspartate aminotransferase and alanine aminotransferase levels were more than twice the upper limit of normal, even though the maximum drug dose was administered.

Treatment block was defined as the change of medication or the end of the observation period, and the duration of each treatment block was tracked until drug discontinuation or the end of the study period.

### Analysis of treatment effectiveness and adverse effect

Kaplan–Meier analysis was used to examine the reasons for drug changes resulting from adverse drug effects and ineffectiveness. Other factors influencing drug changes, such as patient requests or drug unavailability, were also censored. We specifically analyzed cases of monotherapy involving the three drugs, excluding those involving a combination of zinc and chelators from the analysis. *P*-value was determined using the log-rank test (Mantel-Cox test).

A univariate Cox regression model was used to assess the relation of sex, age at diagnosis, age at the start of treatment, and liver cirrhosis at the time of diagnosis with drug discontinuation resulting from adverse drug effects and ineffectiveness. If the *p*-value was found to be less than 0.2, a multivariate Cox regression model was performed using backward selection. In the analysis, statistical significance was defined as a *p*-value ≤ 0.05.

## Results

### Patients

Ninety-two patients diagnosed with WD were included in the evaluation, of whom 46 were female (50%) (Table 1). The median age at diagnosis was 8.3 years (interquartile range [IQR] 5.8–13.2), and the average follow-up period was 7.9 years (IQR 2.0–14.9). Among the 86 patients (93.5%) presenting with hepatic symptoms, 64 were diagnosed incidentally through blood tests without exhibiting any symptoms related to liver disease. Two patients presented with neurological symptoms, and four patients had a mixed presentation of both liver and neurological symptoms.

**Table 1:** Characteristics of the study population

Total patients	
Number of patients	92
Sex	
Male (%)	46 (50.0)
Female (%)	46 (50.0)
Age at diagnosis (y), median (IQR)	8.34 (5.8–13.2)
Clinical manifestation	
Hepatic (%)	86 (93.5)
Neurologic (%)	2 (2.2)
Mixed (%)	4 (4.3)
K-F ring at diagnosis (%)	15 (16.3)
Liver cirrhosis at diagnosis (%)	15 (16.3)
Fatty liver at diagnosis (%)	20 (28.1)
Splenomegaly at diagnosis (%)	15 (16.3)
Abnormal brain MRI finding at diagnosis (%)	7 (7.6)
Acute liver failure at diagnosis (%)	7 (7.6)
Liver transplantation (%)	4 (4.3)
Death	0
First-line medical therapy	
Number of patients	90
D-penicillamine (%)	25 (27.8)
Trientine (%)	24 (26.7)
Zinc (%)	33 (36.6)
Zinc + chelator (%)	8 (8.9)
Patients with medication Changes	
Number of patients	90
No (%)	37 (41.1)
1 time (%)	14 (15.6)
2 times (%)	16 (17.8)
3 times (%)	13 (14.4)
4 times (%)	6 (6.7)
5 times (%)	4 (4.5)

*Abbreviations*: *K-F* Kayser–Fleischer rings

Of the patients with hepatic presentation, seven were diagnosed with acute liver failure, and four of them received liver transplantation (LT) during their initial admission. As two of these patients underwent immediate LT without medication treatment, they were subsequently excluded from further analysis, including Kaplan–Meier analysis.

**Fig. 1:**
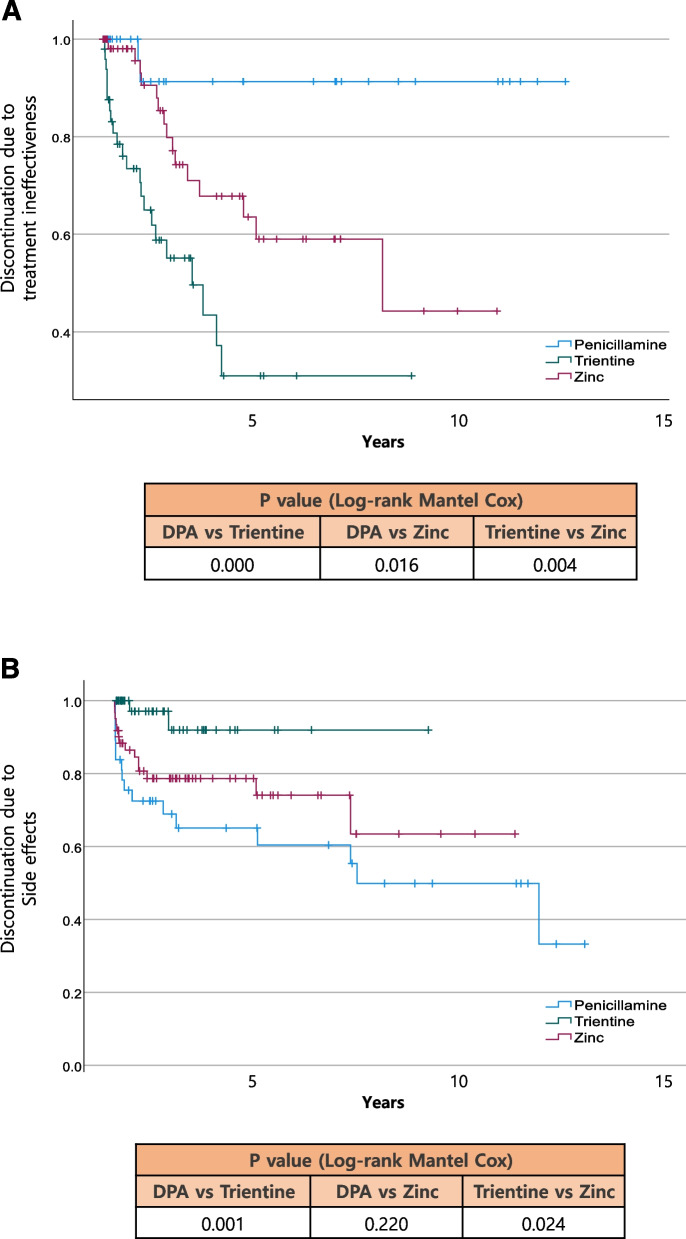
**A** Discontinuation of treatment due to treatment ineffectiveness. **B** Discontinuation of treatment due to adverse effects

Among the 90 patients who received medication, 37 remained on the same medication without any changes. Fourteen patients changed their medication once, while 16, 13, 6, and four patients switched their medication two, three, four, and five times, respectively (Fig. 1A).

Of the 225 treatment blocks, 23 cases involving combination therapy and 54 cases in which changes were not attributed to ineffectiveness or adverse effects were excluded. As a result, a total of 148 treatment blocks (37 DPA, 50 trientine, 61 zinc) were included in the analysis.

### Discontinuation of treatment due to treatment ineffectiveness

When treatment changes were made due to inadequate drug efficacy, trientine was the most frequently chosen option in 22 of 50 cases (44%), followed by DPA in two of 37 cases (5%), and zinc in 15 of 61 cases (25%). Statistical significance was observed when comparing trientine with both DPA and zinc (*p* < 0.5). Similarly, a significant difference observed between DPA and zinc (Fig. 1A).

### Discontinuation of treatment due to drug adverse effect

Out the of 37 patients in the DPA group, 16 (45%) changed their medications owing to drug adverse effects. In the trientine group, two of 50 patients (4%) experienced medication changes due to adverse effects. In the zinc group, 14 out of 61 patients (23%) switched medications due to adverse drug effects.

DPA exhibited a significantly higher incidence of adverse effects compared to trientine (*p* = 0.001). However, no statistically significant difference was observed between DPA and zinc (*p* = 0.220). The proportion of cases in which zinc medication changes were prompted by adverse effects was significantly higher than that of trientine (*p* = 0.024) (Fig. 1B).

The types of adverse effects associated with the three drugs and the time of onset after treatment initiation were categorized into three periods: within 4 weeks, within 1 year, and after 1 year. The details are presented in Table 2. Of the 16 patients who experienced adverse reactions to DPA, six switched their medication within 4 weeks, and six did so within 1 year of starting treatment. Hair loss was reported in two patients after 15 and 8 years of DPA use, respectively. One patient developed idiopathic thrombocytopenic purpura after 8 years of use. Another patient experienced vasculitis after approximately 5 years of treatment, which resulted in a permanent condition requiring ongoing treatment. There were two cases of discontinuation of the drug due to adverse effects of trientine: double vision after taking trientine for 6 months and twisting movements of the arm after taking it for 2 years. Fourteen patients experienced adverse effects of zinc, and all had gastrointestinal symptoms such as abdominal pain, nausea, and heartburn. In one patient with DPA-induced vasculitis, ongoing treatment was required as a permanent condition, and in all other patients, symptom related adverse effects improved after discontinuation of the drug.

**Table 2:** Details of side effects and number of patients according to the duration of drug administration

Onset of side effects after treatment initiation	DPA (*n* = 16)	Trientine (*n* = 2)	Zinc (*n* = 14)
< 4wks	Rash, 3 Pancytopenia, 2 Fever, 1		Abdominal pain, 2 Nausea, 3
< 1 year	Rash, 2 Nausea, 1 Proteinuria, 2 Breast enlargement, 1	Double vision, 1	Abdominal pain, 4 Nausea, 2
> 1 year	Hair loss, 2 Vasculitis, 1 Idiopathic thrombolytic purpura, 1	Twisting movements of the arm, 1	Heartburn, 1

### Discontinuation of treatment due to any cause

Regarding the overall discontinuation of therapy, regardless of the reasons, there was no significant difference between the three drugs (all *p* > 0.05).

### Determinant of drug survival due to any cause

Cox regression analysis was used to examine the association between sex, age at diagnosis, age at the start of drug treatment, liver cirrhosis at diagnosis, and drug discontinuation. However, the analysis revealed that none of these four factors showed a significant relationship with drug discontinuation.

## Discussion

WD is a genetic disease that can be treated. The early initiation of medication is crucial to prevent complications arising from the accumulation of copper in the liver and other organs. In the past, the prevalence of WD was estimated to be approximately 30 per 1 million people [12]; however, with the rise in genetic testing for diagnosis, there has been an increase in the rate of early detection. Recent reports from Hong Kong indicate an annual incidence rate of 1.4 per 1 million people [13], while in Thailand, the annual incidence was reported to be 2.7 per 1 million people [14]. A recent study conducted in Korea utilized the National Health Insurance Service (NHIS) database from 2010 to 2016. The findings revealed an annual incidence rate of 3.8 per 1 million people for WD, with 43.1% of patients diagnosed before the age of 20 years [15]. Consequently, there is a pressing need for active research focused on the diagnosis and treatment of WD in children across Asia. However, to date, there is a scarcity of comparative studies examining the efficacy and potential adverse effects of WD treatment, particularly in the pediatric population.

Over the past two decades, guidelines regarding the diagnosis and management of WD have been published [9, 16–20]. For pediatric patients, European guidelines recommend using DPA and trientine as the initial treatment for children with significant liver disease, such as cirrhosis or abnormal INR, whereas zinc therapy is recommended for presymptomatic patients or as maintenance therapy after decoppering with chelators [19]. However, due to a lack of consensus on defined criteria for clinical classification of WD, guidelines cannot be universally applied to individual patients. As a result, the management of WD patients globally exhibits heterogeneity [21].

DPA has been used as a treatment for WD since the 1950s [22]. However, it is associated with known adverse effects that occur in approximately 30% of patients [23–25]. Notably, owing to the potential for significant neurological deterioration as a serious adverse effect, DPA is not recommended as a treatment option for patients presenting with neurological symptoms [26–29].

In the present study, the incidence of adverse effects was relatively high (approximately 45%) in the patients treated with DPA. However, among the 16 patients who experienced adverse effects, only one case of irreversible complications was reported. The patient was diagnosed with WD at 5 years of age and switched to DPA at 8 years of age. Despite previous treatment with trientine and a combination of trientine and zinc, no improvement in liver function was observed. After taking DPA for 5 years, the patient developed symptoms, such as pulmonary hemorrhage, microscopic hematuria, and proteinuria, at approximately 13 years of age. Subsequent kidney biopsy confirmed pauci-immune crescentic glomerulonephritis, leading to diagnosis of D-penicillamine-induced ANCA-associated vasculitis [30].

In this study, only two patients had neurological symptoms as the initial symptom of the disease, and DPA was not used as the first treatment for these patients. DPA administration was started at 5–10 mg/kg or 150–300 mg/day, and the dose was gradually increased to 20 mg/kg while monitoring the effects and side effects. None of the patients taking DPA developed new neurological symptoms while taking the drug. Although DPA had a relatively high incidence of adverse effects, it was more effective than other drugs. Among the patients treated with DPA, only two out of 37 (5%) showed no improvement and required a change in medication. Initially, DPA was the first-line treatment for both patients, but it was subsequently switched to trientine. However, even with trientine, there was no improvement in drug efficacy, necessitating a change in treatment to a combination therapy involving zinc. Weiss et al*.* compared chelator and zinc monotherapy in the study involving 288 adults with WD. The treatment effect of chelator was significantly greater than that of zinc, but there was no significant difference in adverse effects sufficient to change the treatment [10]. A recent systematic review examining the treatment of WD in both pediatric and adult patients reported similar treatment effects between DPA and zinc, but with a higher occurrence of adverse effects associated with DPA [31]. Patients treated with DPA showed a significantly higher frequency of neurological deterioration compared to those treated with zinc (RR: 1.96, 95% CI: 1.31%–2.93%, *p* = 0.001). It is important to exercise caution when interpreting the therapeutic effect of the drug, as the studies included in the systematic review employed varying doses of DPA and zinc.

In 1969, trientine was introduced as a second-line treatment for adverse effects of DPA [32, 33]. Currently, it is used as first-line treatment along with DPA for symptomatic patients with WD, with trientine being the preferred option in the presence of neurological symptoms [8]. In this study, only two patients experienced reversible adverse effects. Among the 50 patients who received trientine, 22 switched to another medication due to its low therapeutic efficacy. However, during the follow-up period, six of the 22 patients were re-treated with trientine. Among them, three patients demonstrated a positive therapeutic response to trientine and continued with trientine monotherapy. Considering the potential changes in copper absorption, metabolism, and pharmacokinetics with age among pediatric patients, further studies on sequential therapy in WD will be necessary.

Zinc is recommended as a maintenance treatment for symptomatic patients with WD and as a first-line treatment for asymptomatic patients [9]. In this study, all of the adverse effects associated with zinc were gastrointestinal symptoms. Previous studies have reported additional adverse effects of zinc, including copper deficiency-related anemia, neutropenia, sensorimotor neuropathy, myelopathy, and worsening of neurological symptoms [34–37]; however, in this study, there were no adverse effects other than gastrointestinal symptoms. Zinc treatment is generally considered relatively tolerable in children [38–42]. Importantly, this study found that zinc was significantly more effective than trientine, a chelator, in treating WD. Therefore, zinc therapy can be effectively utilized in the early diagnosis and treatment of pediatric WD.

This study had some limitations inherent to retrospective research. The study included five patients out of the seven diagnosed with ALF, excluding the two patients who underwent emergency LT. Among the included patients, three had been receiving medical treatment for more than 2–6 years and were under observation. The remaining two patients had an INR over 2.0 and bilirubin levels ranging from 2 to 5 mg/dL at the time of diagnosis. Despite receiving inpatient treatment for 2 weeks and 3 weeks, respectively, there was no improvement in liver function, leading to the decision to undergo LT [43]. One of the two patients developed pancytopenia during DPA therapy and was included as a case of DPA adverse effects in the Kaplan–Meier analysis. However, these patients may be challenging to assess for treatment response, which could be considered a limitation of the study design. Another limitation of our study is the low proportion of treatment blocks used in the analysis, and the short duration of each treatment. This is due to many cases of medication changes due to lack of drug stock and improved treatment effects, such as changes from chelators to zinc or from combination therapy to monotherapy. Our study aimed to comprehensively understand the reasons for medication changes in the actual treatment of pediatric WD patients without considering the duration of medication administration. Since drug adverse effects can appear abruptly within a month or even a week, this study is thought to be more helpful in interpreting the impact of drug adverse effects on maintenance therapy in pediatric WD patients compared to adult studies that only include statistical analysis of cases with a medication duration of six months or more [10). Despite the retrospective study's limitations, this is the first study to compare the treatment effectiveness and adverse effects of DPA, trientine, and zinc in the real-world practice of pediatric WD patients.

In conclusion, DPA, zinc, and trientine have therapeutic effects in that order, but DPA and zinc each have more adverse effects than trientine in pediatric WD.

## Data Availability

The datasets of the study are not publicly available due to patient privacy and confidentiality. Anonymized data can be made available from the corresponding author upon reasonable request.

## Abbreviations

DPA: D-penicillamine
WD: Wilson disease
LT: Liver transplantation
